# New method to calculate the dynamic factor–flow velocity in Geomorphologic instantaneous unit hydrograph

**DOI:** 10.1038/s41598-019-50723-x

**Published:** 2019-10-02

**Authors:** Yingbing Chen, Peng Shi, Xiaomin Ji, Simin Qu, Lanlan Zhao, Fengcheng Dong

**Affiliations:** 1 State Key Laboratory of Hydrology-Water Resources and Hydraulic Engineering, Hohai University, No. 1 Xikang Road, Gulou District, Nanjing, 210098 China; 2 State Key Laboratory of Hydrology-Water Resources and Hydraulic Engineering, Hohai University, No. 1 Xikang Road, Gulou District, Nanjing, 210098 China; 3 Jiangsu Province Hydrology and Water Resources Investigation Bureau, No. 5 Shanghai Road, Gulou District, Nanjing, 210098 China; 4 State Key Laboratory of Hydrology-Water Resources and Hydraulic Engineering, Hohai University, No. 1 Xikang Road, Gulou District, Nanjing, 210098 China; 5 Information Center (Hydrology moniter and forecast center), MWR, Beijing, 10053 China; 6 State Key Laboratory of Hydrology-Water Resources and Hydraulic Engineering, Hohai University, No. 1 Xikang Road, Gulou District, Nanjing, 210098 China

**Keywords:** Environmental sciences, Hydrology

## Abstract

The determination of characteristic flow velocity is a hydrodynamic problem needs to be solved in the application of geomorphologic instantaneous unit hydrograph (GIUH) for runoff simulation in areas with no or limited data. In this study, 120 watersheds are collected to construct a regression model; 85 of these basins are used for regression analysis, and the 35 remaining basins are utilized to verify the feasibility of the constructed model. Random forest algorithm is applied to screen out important geomorphologic factors from the 16 extracted factors that may affect flow velocity. Multivariate regression is used to establish the numerical relationship between velocity and the selected factors. Sensitivity analysis of each adopted factor in the constructed model is conducted using the LH-OAT method. The rationality and feasibility of the regression model are validated by comparing the flow velocity calculation with a previous approach, which is also calculated based on geomorphological parameters. Subsequently, the runoff simulation based on the GIUH model is evaluated using the proposed technique. Results demonstrate that the proposed formula possesses high fitting accuracy and can be easily used to calculate flow velocity and generate GIUH.

## Introduction

Flood is a natural disaster that severely threatens human lives and the safety of properties^1^. Numerous places all over the world have insufficient hydrologic monitoring equipment, thereby resulting in the problem of observation data scarcity. Flood forecasting in ungauged regions has always been a challenging issue. In 2003, the International Association of Hydrological Sciences launched a decade-long plan for conducting predictions in ungauged basins^2–4^. Accordingly, the rainfall–runoff calculation method in ungauged or less-gauged areas has attracted attention in research on runoff generation and concentration theory, which is also an essential problem faced in the study of flash flood disasters in small and medium watersheds. Seeking a runoff simulation method that is independent of historical hydrological data but has sound physical meaning is the key to solving the aforementioned problems. In the 1960s, Shreve developed an ordered system of geomorphologic elements and discovered that the flow concentration process is induced not only by precipitation characteristics^5,6^, but also by the terrain and topographic conditions of the watershed, thereby indicating a causal relationship between geomorphology and runoff confluence. Given these major theories, the concept of geomorphologic instantaneous unit hydrograph (GIUH) is preliminarily proposed by Rodriguez-Iturbe^7^, Valdes^8^, and Gupta^9^. GIUH describes the movement of each droplet by using the probability statistics method based on the Horton–Strahler (H–S) stream ordering scheme^10–12^ and by determining the effect of basin geomorphology on flow concentration.

Some hydrologists use the Nash model in GIUH generation based on the conceptual cascade model of equivalent linear reservoirs developed by Nash^13,14^, thereby expanding GIUH theory into an intuitive and applicable direction^15,16^. Renzo Rosso initially discovered that parameters “a” and “k” in the Nash model are dependent on the H–S ordering scheme ratios and successfully derived the GIUH as a Nash model form, simplifying the calculation of (instantaneous unit hydrograph) IUH^17^. Roger Moussa derived an equivalent calculation method of the Nash model parameters to generate GIUH to eliminate the impact of area threshold in extracting DEM (Digital Elevation Model) data^18^, which are essential for the extraction of geomorphologic factors. As for the practical application in the data-scarcity Ellouze-Gargouri proposed a method using Monte Carlo simulation and Copula function to estimate the effective rainfall then applying GIUH into ungauged basins^19^; Jaiswal *et al*. developed a geomorphology trial based on a regional Nash model in Central India, and evaluation indexes indicate that the simulation is well performed^20^; Anil Kumar compared the H–S stream-order ratio-based model with Nash’s two-parameter gamma distribution-based conceptual model to derive GIUH and analyzed the performance response of these two types of GIUH^21^. Nevertheless, an insufficient aspect of GIUH generation remains, which depends on the calculation of the hydrodynamic factor and flow velocity yet hinders the practical application of GIUH theory on runoff simulation. The value of flow velocity is often given in empirical way among previous studies^22,23^. Many hydrologists identified this issue and began to investigate valid solutions. In the 1980s^24^, Rodriguez-Iturbe *et al*. proposed a derivation of geomorpho-climatic instantaneous unit hydrograph (GCIUH), which is an improvement of the GIUH, to eliminate the velocity from the results by exploring the functional relationship between flow velocity and effective rainfall intensity and duration. In 1997, Bhaskar *et al*. investigated the relationship between velocity and effective rainfall intensity to make the dynamic parameter easily accessible^25^. Furthermore, they derived a series of empirical formulae and verified their feasibility. The estimation of Manning’s roughness coefficient is commonly used in the velocity calculation in some research^26–28^. However, the effective rainfall intensity and average roughness of ungauged basin are always unavailable. Agnese *et al*. introduced an empirical formula to calculate the travel time of a droplet^29^. However, the equation needs to be changed when the basin changes, which is infeasible in many practical cases. In 2011, Jotish^30^ employed the empirical Kirpich formula^31^ to derive an equation for calculating the time of flow concentration based on the easily measurable watershed parameters of stream length and watershed slope to estimate flow velocity. However, the acceptability and feasibility of the proposed method still need to be determined because the proposed method was tested in only one watershed in India. This technique is employed in this study as a contrast to analyze rationality.

This study aims to determine the internal relation between the characteristic flow velocity and the terrain and geomorphologic features of a watershed and convert it into a numerical and functional form by using the multivariate regression method. A total of 120 sub-basins in the Yangtze River Basin were studied to propose a convenient and applicable approach of generating the GIUH into hydrologic process simulation.

## Background

Nash^13,14^ derived a mathematical formula of the unit hydrograph (UH), which contains fewer parameters but is applicable in practical conditions based on the hypothesis that the effects of watershed storage can be simulated by several linear reservoirs in series connection. The formula is expressed as follows:

1 $${\rm{u}}({\rm{t}})={(\frac{t}{k})}^{a-1}\frac{{e}^{-\frac{t}{k}}}{k\Gamma (a)},$$

where u(t) stands for the UH ordinate; index *a* is the shape parameter that reflects the number of conceptual free water reservoirs; and *k* is the scale parameter, which is a constant reservoir storage that delineates the average watershed flow time of concentration. Γ(*a*) is the gamma function of *a*. In previous literature^17^, the shape parameter *a* relies on H–S geomorphologic characteristics *R*_*A*_, *R*_*B*_, and *R*_*L*_^10–12^ of a watershed, and *k* depends not only on geomorphology but also on the flow velocity. Croley applied a numerical solution to obtain *a* against *R*_*A*_, *R*_*B*_, and *R*_*L*_ as Eq. (2), and the normal ranges of H–S geomorphologic numbers from 3 to 6, 2.5 to 5, 1.5 to 4.1 are proposed by investigating the features of a natural river network^32^.

2 $$a=3.29{({R}_{B}/{R}_{A})}^{0.78}{{R}_{L}}^{0.07},$$

3 $${\rm{k}}=0.70(\frac{{R}_{A}}{{R}_{B}{R}_{L}}){}^{0.48}\frac{{L}_{\Omega }}{v}.$$

where ${{\rm{L}}}_{\Omega }(km)\,$ is the highest-order river length, and *v*(*m*/*s*) represents the hydrodynamic factor flow velocity; *R*_*A*_, *R*_*B*_, and *R*_*L*_ represent the area, bifurcation, and length ratios of a catchment, respectively. Parameters *R*_*A*_ *R*_*B*_, *R*_*L*_, and *L*_Ω_ can be easily extracted from the DEM data, whereas the flow velocity *v* is always difficult to obtain. Velocity is the focus of this study.

Flow velocity is a dynamic factor and a key parameter in the GIUH-based model^30^. In the process of seeking an appropriate and reasonable way to calculate flow velocity, some hydrologists and geomorphologists attempted to determine the numerical relation between velocity and catchment geomorphology. In previous literature^33–35^, different empirical flow velocity calculation methods related to Manning’s roughness are proposed and employed, such as

4 $$v={\rm{a}}\sqrt{{\rm{S}}},$$

where a is a parameter related to Manning’s roughness coefficient, and S is the average slope of the watershed. Equations, such as the Eagleson–Bras formulas^36^, contain the rainfall intensity

5 $$v=0.665{\alpha }_{\Omega }^{0.8}{(i{A}_{\varOmega })}^{0.4},$$

6 $${\alpha }_{\Omega }={{\rm{S}}}_{c}^{1/2}/n{B}^{2/3},$$

where *i* is the rainfall intensity, cm/h; *A*_Ω_ is the basin area, km^2^; S_c_ is the channel slope; B is the average width of river, m; and n is Manning’s roughness. However, these formulas have considerable uncertainty, that is, rainfall intensity is barely accessible in ungauged areas. Moreover, coefficients that are related to Manning’s roughness, which are often dependent on the experience of the observers, are difficult to obtain. Thus, employing these formulas in real applications is difficult. An equation was derived to calculate velocity from the well-known Kirpich formula by Jotish^30^, which also relies on geomorphologic features

7 $${t}_{c}=0.01947{L}^{0.77}{{\rm{S}}}^{-0.385},$$

8 $${t}_{c}=(L/v)/60,$$

9 $${v}_{k}=0.8562{L}^{0.23}{{\rm{S}}}^{0.385},$$

where *t*_*c*_ is the time of flow concentration, min; *L* is the length of the main stream, m; S is the mean slope of catchment, m/m; and *v*_*k*_ is the hydrodynamic parameter velocity, m/s.

In this study, Eq. (9) is used to calculate the average flow velocity as a contrast to analyze the fitting degree and the rationality.

### Study areas and data preprocessing

**Figure 1:**
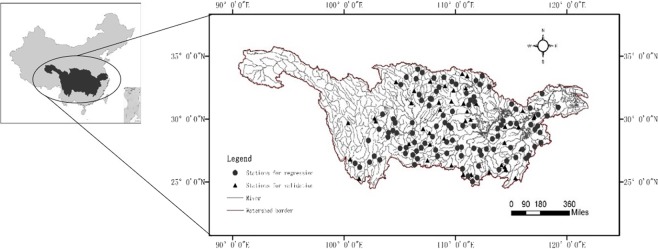
Location of the Yangtze River Basin in China and distribution of the 120 study watersheds control stations.

The research areas locate in the Yangtze River Basin in China, which is the third largest river basin in the world. A total of 120 mutually non-nesting watersheds (drainage area from 35.3 km^2^ to 7,289 km^2^) and their corresponding basic characteristics in these catchments are collected and listed in Appendix I to reduce the considerable impact of human activities on the flow concentration process, which may alter the reliability of results, and to investigate a relatively pure numerical relationship between geomorphological parameters and hydrodynamic parameter velocity. Figure 1 shows the distribution of the control stations in the 120 catchments (85 for regression model construction and 35 for validation).

The Yangtze River Basin (1.8 million km^2^) stretches across China from the west to the east, accounting for 18.8% of the total land area of China. The terrain and geomorphology in the Yangtze River Basin are complex and resemble a multi-step ladder shape due to the vast drainage area. Thus, different types of terrain, such as mountains, plateaus, and plains, are contained in this basin. The temporal and spatial distribution of precipitation is uneven. The annual average precipitation in the Yangtze River Basin is 1,067 mm. However, the source part (the west part) of the basin belongs to the arid region, where the multi-year average precipitation is less than 400 mm. The Western Sichuan Plateau, Qinghai, Gansu, and the northern part of the middle reaches of Yangtze River are mainly located in semi-humid zones with an annual precipitation from 400 mm to 800 mm. The south part of the middle reaches and the lower Yangtze River Basin are generally located in the humid area an average annual rainfall between 800 and 1,600 mm. In some mountainous areas, the average annual rainfall is even higher than 1600 mm.

Generally, the evaporation of the land surface decreases with the increase in elevation, that is, a high elevation corresponds to a low land evaporation. The average annual land surface evaporation is 541 mm, and the spatial distribution shows a trend in which the middle and lower reaches are larger than the upper streams, the plains and basins are larger than the mountains, and the south bank is larger than the north.

The basic requisite data, including observed precipitation, evaporation, and flow discharge data, are acquired from the hydrologic yearbook of the Yangtze River Basin. The characteristic values of geomorphology and terrain, including the stream slope, the river length, and the H–S ratios of each sub-basin, are extracted based on the DEM data at the resolution of 30 m × 30 m. The streams are ordered based on the H–S ordering scheme. In each watershed, more than 30 historical rainstorms, including the rainfall from the rainfall station and the discharge records from the hydrologic station on the outlet, are excerpted.

## Methodology

### Optimal flow velocity acquisition

As previously mentioned, more than 30 historical flood event data are collected in each watershed to obtain the optimal flow velocity, ${v}_{o}$, by using the optimal method and Eqs (1–3). An automatic calibration program was developed for this purpose. With the use of the program, flow velocities from 0.1 to 10 are inputted at a 0.01 step to generate the corresponding GIUH and subsequently simulate the runoff process. The optimization method is applied by setting the highest Nash–Sutcliffe efficiency (NSE) coefficient in the flood simulation as the objective function to acquire *v*_*o*_ for each basin.

### Significant factor selection

Sixteen topological and geomorphological parameters, namely, Ω-order river length (L_Ω_), drop of Ω-order river (D_Ω_), the longest river (L_*m*_), basin area (A), the number of river source (S), river bend degree (D_*bend*_), river bend degree ratio (R_*bend*_), bifurcation ratio (R_*B*_), difference between the highest and lowest elevations (relief), area ratio (R_*A*_), river length ratio (R_*L*_), relief ratio (RR), river network density (Density), river network density ratio (Rs), ratio of each order main stream (R_Ω_), and drop ratios of each order river (*R*_*drop*_), are extracted from the DEM data and collected for each watershed by screening the important factors.

The relationship between the topographic feature values and the characteristic flow velocity is complex. Thus, the model factors should be selected comprehensively. The random forest (RF) algorithm is a suitable for handling the selection and classification work and is commonly used in different fields^37–39^. In this study, the RF model is built to screen out the core factors.

#### RF introduction

The RF algorithm is a burgeoning machine learning method with high flexibility and reliability and is suitable for multivariate big data processing. RF is a classifier that consists an entire forest of uncorrelated trees applying a procedure similar to CART modeling, combined with randomized node optimization and bagging method^40^. Many studies^41,42^ reported that the RF algorithm can handle noise and outliers well with low generalization error and obtain good estimation and prediction results that are insensitive to multivariate collinearity. Mtry (trial times) and Ntree (number of trees) are two important factors that affect the performance and efficiency of the RF model. A small Mtry value can result in the overfitting of the tree classifier, increased error in classification prediction, and reduced accuracy. Conversely, this value affects model building and running speed. If Ntree is small, then training is insufficient and the randomness of RF is reduced; otherwise, it will be over stochastic that the computational amount of the model increases while the classification accuracy of the tree classifiers is reduced. Therefore, the optimal values of Mtry and Ntree are essential in the algorithm training, which is aided by R language software.

#### Importance measurement

Two different aspects of metric importance calculation are used in RF. One of the evaluation indicators is the average incline percentage of the mean square error (MSE) (%IncMSE). MSE is defined as follows:

10 $${\rm{MSE}}=\frac{1}{N}{\sum }_{i=1}^{N}{({y}_{i}-{\hat{y}}_{i})}^{2},$$

where N is the number of variables, and ${\hat{y}}_{i}$ is the *i* th predictive value of the observative *y*_*i*_.

The other indicator is the average decrease in node purity (IncNodePurity), which is equivalent to the average decrease in Gini impurity. The calculation formula of the Gini impurity value is presented as (11).

11 $${\rm{Gini}}(t\,)=1-{\sum }_{j=1}^{k}{[p(j|t)]}^{2},$$

where m, n, and t represent the number of metrics, tree classifiers, and nodes in a tree, respectively.

### Sensitivity analysis

Global sensitivity analysis (GSA) increasingly evolved in the assessment of hydrological models due to the development of computer technology^43^. In the previous literature and corresponding application, many different GSA methods have been compared and proposed in recent years^44–46^. LH-OAT, RSA, and GLUE are commonly used model sensitivity analysis techniques. In this paper, the LH-OAT method^47^ is applied to analyze the sensitivity of parameters in the constructed model, and it maintains the robustness of the LH method and the accuracy of the OAT method. Loop work is operated in LH-OAT, that is, for parameter i in sample j, the relative sensitivity index *S*_*ij*_ is calculated from (12,13)

12 $${S}_{ij}=|\frac{100\ast (\frac{f({\theta }_{1},\ldots {\theta }_{i}\ast (1+{\Delta }_{i}),\ldots ,{\theta }_{P})-f({\theta }_{1},\ldots {\theta }_{i},\ldots ,{\theta }_{P})}{(f({\theta }_{1},\ldots {\theta }_{i}\ast (1+{\Delta }_{i}),\ldots ,{\theta }_{P})+f({\theta }_{1},\ldots {\theta }_{i},\ldots ,{\theta }_{P}))/2})}{{\Delta }_{i}}|,$$

13 $${S}_{i}=\frac{1}{N}{\sum }_{1}^{N}{S}_{ij},$$

where *f*(·) is the objective function; Δ_*i*_ is the noise that adds up to parameter *θ*_*i*_; N is the number of samples; and *Si* is averaged from *Sij* of N samples. A large *Si* corresponds to high sensitivity of *θ*_*i*_.

### Model performance evaluation

As for the model performance evaluation, the graphical and the numerical methods are both presented in this paper. The hydrographs are drawn to compare the predicted series and the observed ones on the time series plot intuitively. Meanwhile, several criteria to measure the goodness-of-fit quantitively are considered, including peak flow, runoff depth and four commonly used numerical criteria: relative peak flow error (RPE), relative runoff depth error (RRE), time-to-peak error (TPE), and Nash-Sutcliffe efficiency (NSE):

14 $${\rm{RPE}}=\frac{({Q}_{Pc}-{Q}_{P0})}{{Q}_{P0}}\ast 100 \% $$

15 $${\rm{RRE}}=\frac{(R{D}_{c}-R{D}_{0})}{R{D}_{0}}\ast 100 \% $$

16 $${\rm{TPE}}={t}_{pc}-{t}_{p0}$$

17 $${\rm{NSE}}=1-\frac{{\sum }_{t=1}^{n}{({Q}_{0}-{Q}_{c})}^{2}}{{\sum }_{t=1}^{n}{({Q}_{0}-\overline{{Q}_{0}})}^{2}}$$

where, *Q*_*Pc*_: calculated peak flow (m^3^/s); *Q*_*P0*_: observed peak flow ((m^3^/s)); *RD*_*c*_: calculated runoff depth (mm); *RD*_0_: observed runoff depth (mm); *t*_*pc*_: time to peak of the calculated series (h); *t*_*p*0_: time to peak of the observed series (h); *Q*_0_: the observed discharge (m^3^/s); *Q*_*c*_: the calculated discharge (m^3^/s); $\,\overline{{Q}_{0}}$: is the mean of observed data *Q*_0_ (m^3^/s); *t*: time interval. It has been proved that NSE is much superior than the deterministic coefficient as a criterion to evaluate the model performance^48^ with the specific ratings^49^:

$${\rm{NSE}} < 0.50\,{\rm{unsatisfactory}};$$

$$0.50 < {\rm{NSE}}\le 0.65\,{\rm{satisfactory}};$$

$$0.65 < {\rm{NSE}}\le 0.75\,{\rm{good}};$$

$$0.75 < {\rm{NSE}}\le 1\,{\rm{very}}\,{\rm{good}}.$$

## Results

### Results of optimal flow velocity

The optimal flow velocity results are automatically calibrated by the developed program by setting the highest goodness-of-fit indices and Nash–Sutcliffe coefficient as goal functions. The results of *v*_*o*_ in some watersheds are listed in Table 1 as examples.

**Table 1:** The optimal characteristic flow velocity (*v*_*o*_) in some sample watersheds.

Watershed	Bainiqiao	Baiyan	Beidou	Caoba	Chenzhou	Dahebian	Qingchuan	Dibao	…
area(km^2^)	210	635	1870	704	361	1274	80.8	512	…
*v*_*o*_(m·s^−1^)	1.63	1.38	2.22	2.56	1.57	2.75	1.31	1.82	…

### Factor selection results

**Figure 2:**
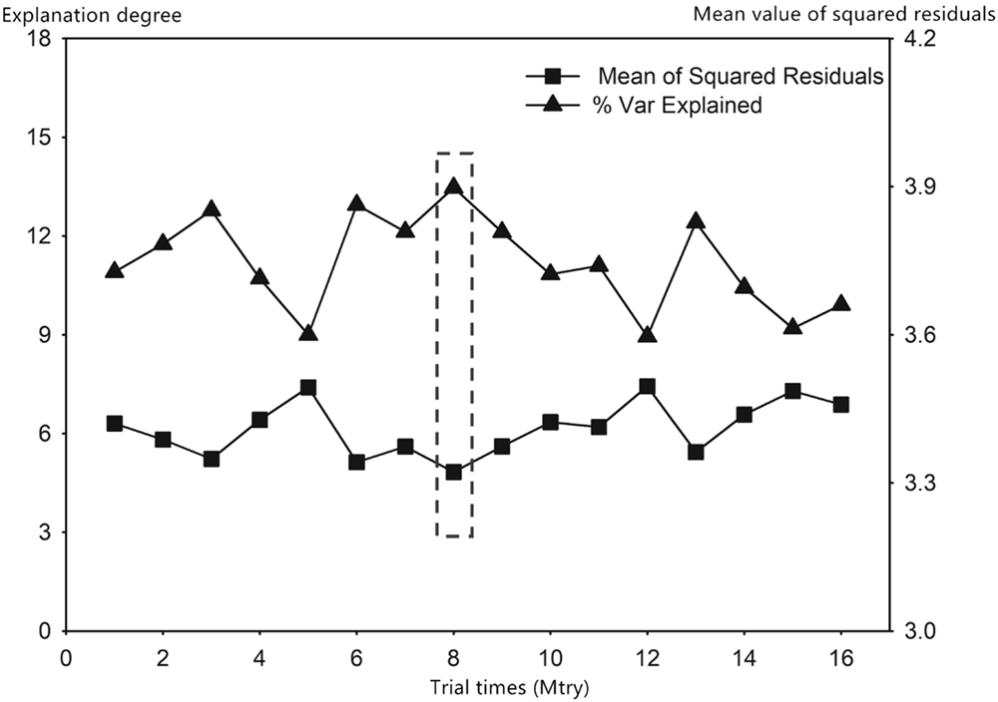
Distribution plot of the Mean of Squared Residuals and explanation degree over the change of Mtry.

**Figure 3:**
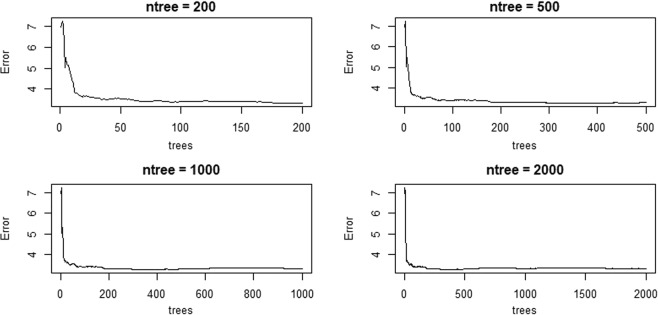
Plots of the relation between model error and Ntree.

**Figure 4:**
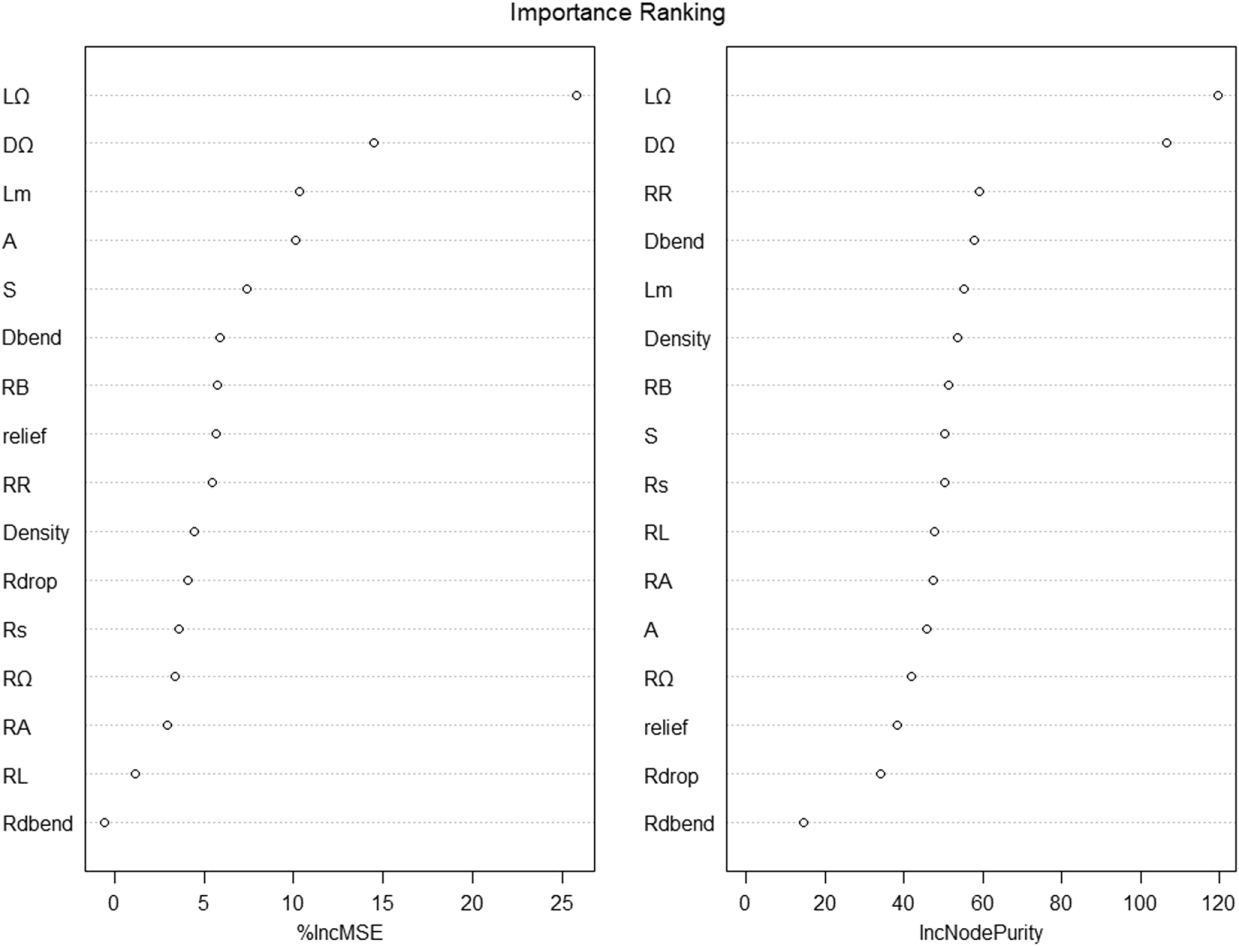
Importance ranking plots of all 16 geomorphological parameters from the RF model.

A RF model was built to screen out the collected geomorphological parameters. Section 4.2.1 shows that the adequate values of Mtry and Ntree are supposed to be assigned at first. Figure 2 shows the mean of squared residuals and the % Var explained (the explanation degree) distribution with a fixed Ntree over the change of Mtry. Noticeably, when Mtry changes, the mean of squared residuals wanes and waxes, which are reversely consistent with the tendency of the explained % Var. Moreover, when Mtry is equal to 8, the mean of squared residuals reaches its maximum, and the explanation degree is minimal. Different Ntree values lead to various model errors. Figure 3 illustrates that when Ntree is approximately assigned as 800, this RF model error tends to be stable. Figure 4 presents the importance of all the geomorphological parameters ranked afterwards based on the aforementioned work. Results show that the percentage of MSE incline and the incline of the node purity L_Ω_ rank first, that is, L_Ω_ most significantly affects flow velocity. In addition, the shape of the IUH can be changed in response to the change in scale, that is, area size is a decisive factor in GIUH generation^7^. Rodriguez also proposed that the size effect and dynamic component should be contained in the waiting time estimation, which is equivalent to the average flow velocity, because velocity equals the result of average waiting time divided by the length of flow path. However, the scale is not dependent on any H–S parameters, which is why the area size is integrated into the model. Thus, Ω-order river length *L*_Ω_ and the watershed area A are taken to participate in the multivariate regression procedure and subsequently into the optimal regression model construction.

### Optimal regression model construction and sensitivity analysis

The numerical relationship between the optimal flow velocity *v*_*o*_ and the two factors can be observed because the critical parameters (A and *L*_Ω_) have been selected out. The numerical model is presented as follows:

18 $${v}_{c}=\mathrm{EXP}\{0.755{(\frac{A}{{{L}_{\Omega }}^{2}})}^{-0.139}\},$$

where *v*_*c*_ is the flow velocity in the constructed model, m/s, which is equivalent to *v*_*o*_.

**Figure 5:**
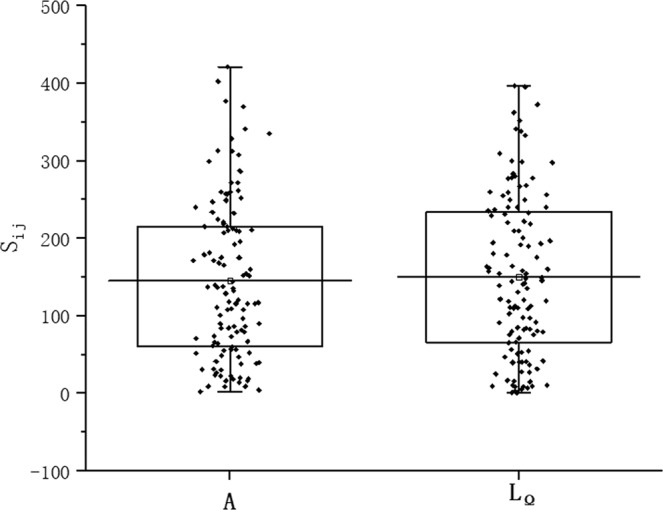
Box plots of the sensitivity index *S*_*ij*_ of A and *L*_Ω_, by the constructed model.

Subsequently, the sensitivity of each parameter in the model is analyzed using the LH-OAT method. The box plot (Fig. 5) shows the sensitivity index distribution of each parameter. A total of 120 points are present on each bar in the box plot, which represent the values of sensitivity index S_ij_ calculated in 120 watersheds. The black lines at the top and bottom of each bar indicate the maximum and minimum values, respectively; the top and bottom lines on the box represent the 75^th^ and 25^th^ percentiles, respectively. The hollow square with a black line in the middle indicates the value of S_*i*_. The plots show that the points on the *L*_Ω_ plot are relatively more densely distributed on the upper part than those on the A plot, and the S_*i*_ value of *L*_Ω_ (150.6) is slightly larger than that of *A* (145.2). These results indicate that parameter *L*_Ω_ is more sensitive than A, which is consistent with the importance ranking of the geomorphological parameters in Fig. 4.

### Validation of the constructed regression model

**Figure 6:**
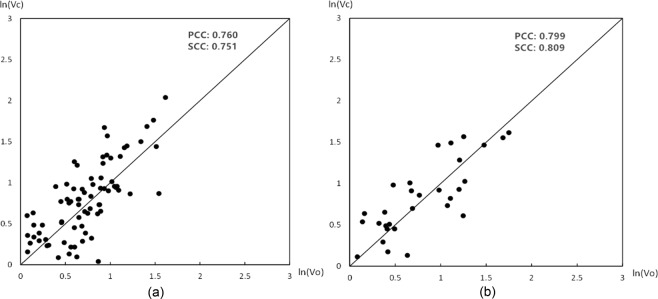
Fitting plots between *v*_*c*_ and *v*_*o*_ at regression stage (**a**) and validation stage (**b**).

The comparison plots between the optimal velocity *v*_*o*_ and the calculated flow velocity from Eq. (14) *v*_*c*_ at the regression stage (85 watersheds) and validation stage (35 watersheds) are curved to verify the fitting degree and testify the rationality of the constructed model (Eq. [14]), as shown in Fig. 6(a,b). Pearson correlation coefficient (PCC) and Spearman’s correlation coefficient (SCC) are calculated to indicate the correlation between *v*_*o*_ and *v*_*c*_. PCC and SCC in the watersheds for regression and validation are 0.760 and 0.751 and 0.799 and 0.809, respectively. Results suggest that the flow velocity calculated from the proposed model fits well with the optimal flow velocity.

**Figure 7:**
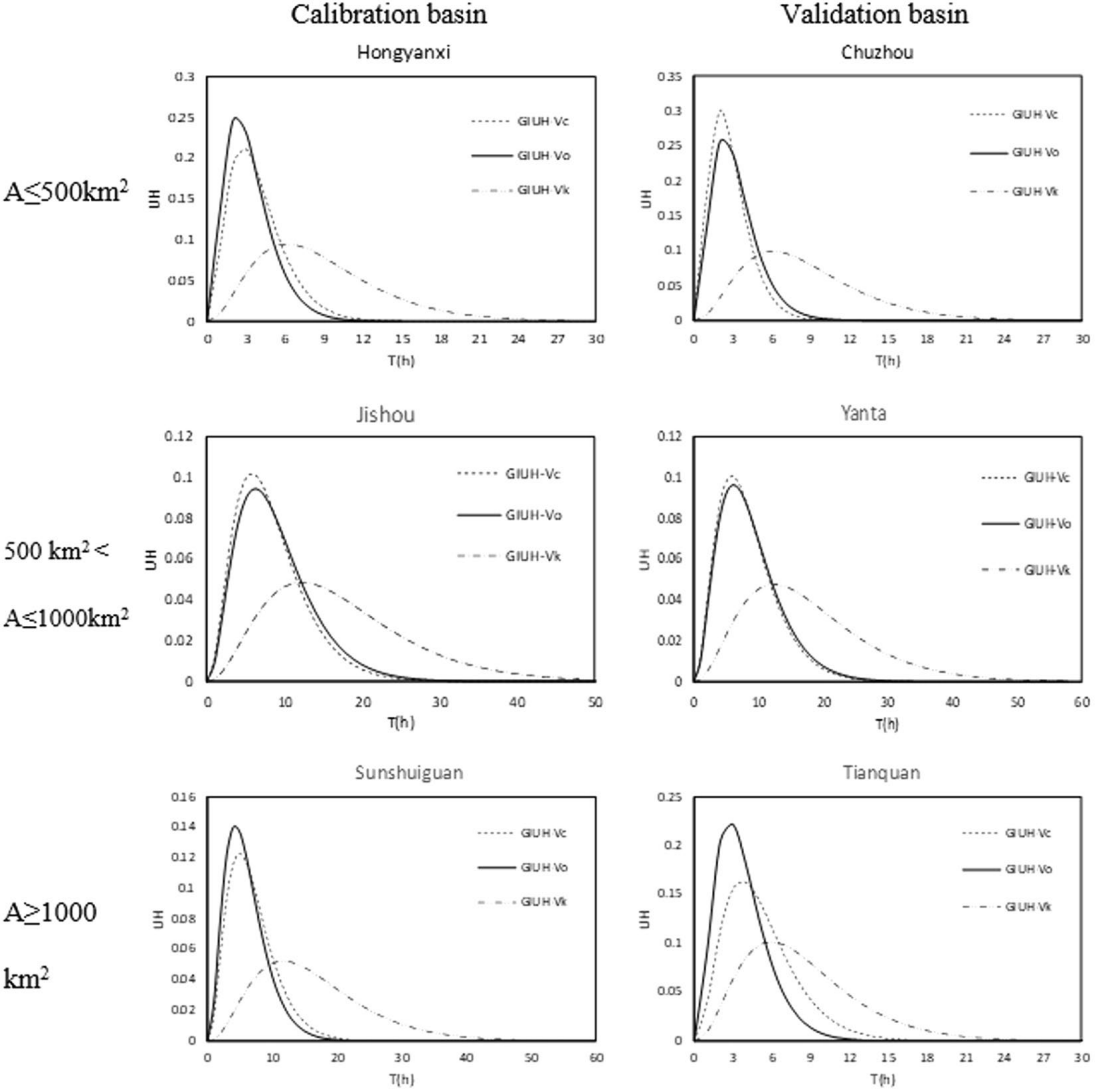
Comparison of GIUHs derived from *v*_*c*_, *v*_*k*_ and *v*_*o*_ in 6 watersheds in different area size.

**Figure 8:**
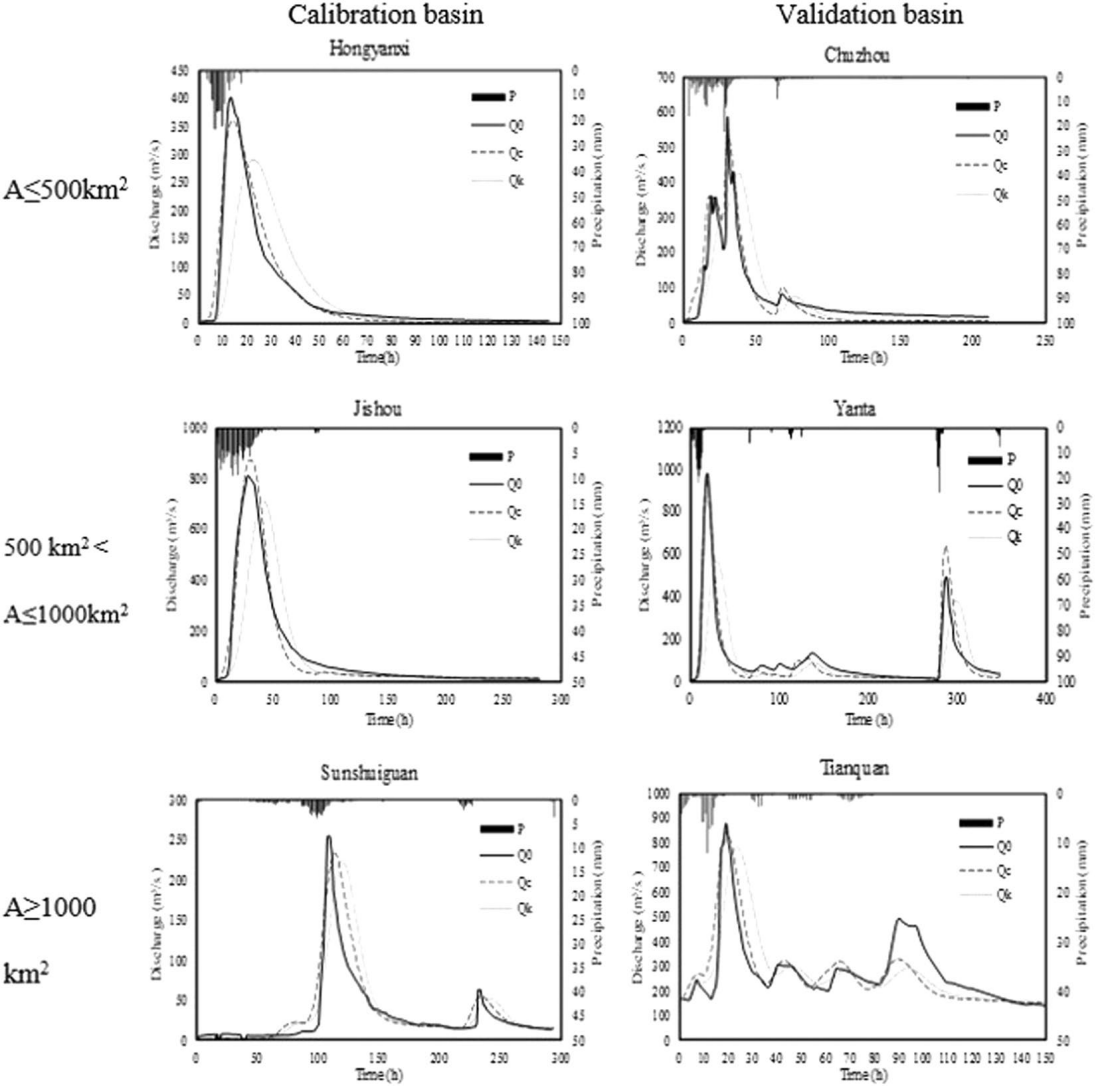
Comparison among the observed and the calculated runoff hydrographs from GIUH–*v*_*c*_ and GIUH–*v*_*k*_ in 6 watersheds.

The practical feasibility of the constructed model is tested in the randomly selected watersheds: three watersheds in different area sizes (A ≤ 500 km^2^, 500 km^2^ < A ≤ 1,000 km^2^, and S ≥ 1,000 km^2^) from each stage (6 in total) are selected, and one flood event is stochastically selected in each catchment. The rationality of the present model is accounted by the comparison among the GIUH generated from *v*_*c*_, *v*_*k*_ (the velocity calculated from Equation [9]), and *v*_*o*_, noted as GIUH–*v*_*c*_, GIUH–*v*_*k*_ and GIUH–*v*_*o*_. The relative error of the IUH peak is calculated to verify the fitting degree of GIUH–*v*_*c*_ to GIUH–*v*_*o*_ and GIUH–*v*_*k*_ to GIUH–*v*_*o*_ respectively, and the results are presented in Table 2. Furthermore, the flood event simulation performance in each test watershed is analyzed. Figure 7 shows the unit hydrograph comparison in these test catchments. Flood simulation based on GIUH–*v*_*o*_, GIUH–*v*_*c*_, and GIUH–*v*_*k*_ and the record runoff process is curved for contrast, as shown in Fig. 8.

**Table 2:** Comparison among *v*_*o*_, *v*_*c*_, and *v*_*k*_, and the UH relative peak error (URPE) results in 6 test basins.

Stage	Basin	*v*_*o*_(m/s)	GIUH–*v*_*c*_		GIUH–*v*_*k*_	
			*v*_*c*_(m/s)	URPE (%)	*v*_*k*_(m/s)	URPE (%)
Regression	Hongyanxi	2.41	2.04	−14.2	0.90	−58.1
	Jishou	1.78	1.92	8.1	0.92	−48.5
	Sunshuiguan	4.40	3.83	−12.3	1.62	−62.9
Validation	Chuzhou	5.03	5.75	17.3	1.90	−61.2
	Yanta	1.57	1.64	4.5	0.77	−50.4
	Tianquan	4.79	3.50	−26.5	2.17	−54.1

Some results after flood calculation and the criteria for the model performance evaluation including peak flow, runoff depth, RPE, RRE, TPE, and NSE are summarized and listed in Table 3. When using GIUH–*v*_*c*_, for all six watersheds at the regression and validation stages, the qualified ratios of RPE, RRE, and TPE simulation are 100%, 83.3%, and 100%, respectively. The absolute value of RPE, RRE, and TPE ranges from 3.0% to 20%, 1.4% to 21.1%, and 0 h to 5 h, respectively. The average NSE value of the six watersheds is 0.871. Accuracy evaluation statistics indicate that the simulation results from GIUH–*v*_*c*_ are generally comparable with the observed flow process for the flood events of regression and validation watersheds. By contrast, the flood process simulated based on GIUH–*v*_*k*_ seem to be inferior to the results of GIUH–*v*_*c*_, of which the average NSE is 0.467. Such results cannot even meet the lowest standard issued in MWR^50^, which is required for flood forecasting or simulation, thereby illustrating that Eqs (7–9) are unsuitable for velocity calculation, at least in the Yangtze River Basin.

**Table 3:** Summarized values of criteria in 6 test watersheds.

Stage	watershed	Area(km^2^)		Peak flow (m³/s)	RPE (%)	Runoff depth (mm)	RRE (%)	TPE (h)	NSE
Regression	Hongyanxi	186	Observed	403		142.66			
			GIUH–*v*_*c*_	361	−10.4	148.34	4.0	1	0.964
			GIUH–*v*_*k*_	293	−27.2	147.87	3.7	9	0.444
	Jishou	775	Observed	812		149.62			
			GIUH–*v*_*c*_	872	7.4	144.37	−3.5	2	0.969
			GIUH–*v*_*k*_	713	−12.4	133.25	−10.92	13	0.607
	Sunshuiguan	1584	Observed	255		20.59			
			GIUH–*v*_*c*_	233	−8.6	24.94	21.1	5	0.776
			GIUH–*v*_*k*_	224	−12.1	24.8	20.4	11	0.499
Validation	Chuzhou	287	Observed	578		188.2			
			GIUH–*v*_*c*_	463	−19.9	187.68	−0.3	1	0.897
			GIUH–*v*_*k*_	428	−25.9	187.66	−0.3	8	0.580
	Yanta	804	Observed	985		169.53			
			GIUH–*v*_*c*_	951	−3.5	166.87	−1.6	−1	0.917
			GIUH–*v*_*k*_	577	−41.4	146.66	−13.5	11	0.209
	Tianquan	1719	Observed	878		94.47			
			GIUH–*v*_*c*_	843	−4.0	93.14	−1.4	0	0.700
			GIUH–*v*_*k*_	781	−11.0	94.39	−0.1	5	0.442

## Discussions

The statistical approach RF model shows that two indexes are employed to judge the importance of 16 geomorphological characteristics. The results from the average increase in the MSE and average decrease in the node purity illustrate that *L*_Ω_ is the most significant factor. Thus, it is included in the model construction. The area size should be considered in a GIUH model building, more specifically, it should be contained in the time of flow concentration estimation because the basin area can influence the flow concentration process, according to the original research of Rodriguez^7^. A most intuitive but logical hypothesis is that the large size of the basin area results in complex catchment attributes, which greatly affect the flow concentration process, such as underlying surface condition and vegetation distribution. Therefore, the use of a single parameter value to generalize each feature is inaccurate. More importantly, a spatially evenly distributed precipitation in a certain area is the presupposition of the application of unit hydrograph method. In other words, for a large basin, considering the rainfall uniformity in spatial scale is irrational. Accordingly, the performance of UH application will be degraded. Thus, *L*_Ω_ and A are integrated into the model construction, thereby keeping the model concise and precise. A relevant indication in the flood simulation results (seen in Fig. 8) of the above analysis shows that the GIUH–*v*_*c*_-based flood simulation of Sunshuiguan and Tianquan, whose basin areas are both larger than 1,000 km^2^, is slightly inferior with an NSE value of 0.776 and 0.700 for the regression and validation stages, respectively. Figure 8(f) shows the second main peak (observed) that mainly leads to the low accuracy of this flood event simulation. The precipitation volume and intensity before this peak flow (during the time interval from 78 to 95) are almost the same as the precipitation from 54 to 71, whereas the runoff response of the latter rainfall is nearly twice the last one. A possible reason for this condition is that the position of the rainstorm center is moving near the outlet. However, in a basin, the rainfall data are measured by precipitation stations, which are in a fixed position. This phenomenon may cause the real precipitation volume (78 to 95) to be actually larger than the observed data from the precipitation stations. Again, this result demonstrates that the basin area is an indispensable factor to the GIUH approach.

In the sensitivity analysis results of the constructed model stated in Chapter 4.3, we found that regardless of the value δ is assigned, L_Ω_ shows higher sensitivity than A in the LH–OAT method. Thus, a random value of δ is set as an example to present the results (Fig. 5). This finding is consistent with the parameter importance ranking results in the RF model. The LH-OAT method is robust and operative and can draw explicit conclusions, such as the significance order of each parameter, while only presenting the sensitivity relation among the factors. The sensitivity index S_*ij*_ is related to δ. Thus, no certain threshold exists to evaluate the sensitivity level. Therefore, if the sensitivity degree of each parameter should be investigated, then the practical application of the model should be explored to obtain a qualitative conclusion.

The rationality of the constructed model is verified in the first step by the comparison between *v*_*c*_ and *v*_*o*_ at the 85 catchments in model regression and 35 catchments in model validation (Fig. 6). In addition, the SCC and PCC for two stages, which exceed 0.750, are calculated. This result indicates a high fitting degree even though the model needs a lot of data. Then, the GIUH-*v*_*o*_, GIUH-*v*_*c*_, and GIUH-*v*_*k*_ of the six test watersheds are all generated and curved in Fig. 8. Notably, the GIUH based on *v*_*c*_ is much closer than GIUH–*v*_*k*_ to GIUH–*v*_*o*_. The UH relative peak errors (URPE) are also summarized (Table 2). For GIUH–*v*_*c*_, only Tianquan watershed is unqualified with the absolute URPE 26.5%. The GIUH–*v*_*k*_ is not ideal for all the test watersheds even though it employs geomorphologic features. The feasibility of the model is examined by conducting a flood simulation comparison between the observed discharge and the discharge calculated from GIUH–*v*_*k*_; this approach was previously discussed.

The proposed model comprehensively considered the important factors that were selected based on statistics method; these factors are easy to obtain and simple to calculate. The relatively high accuracy of the presented flood simulation shows that this approach is feasible and rational for calculating the flow velocity in regions without available data or have data scarcity.

To apply the proposed model with high precision to large-area watersheds, such as Tianquan Basin and Sunshuiguan Basin, the generalized impact from the area scale can be reduced by watershed division. This issue will be expanded based on this study. Several aspects can be further improved and explored to enhance the accuracy of the simulation of the proposed regression formula. First, DEM data are indispensable in the study. However, the area threshold for extracting the basin information to acquire the topography and geomorphology parameters was not considered and can thus be explored in future research. Second, in the natural condition, the relationship between the hydrodynamic factor and the topographic indexes may be complex. The exponential form constructed in this study is commonly accepted to consider the nonlinear relation between the arguments and the dependent variable. However, the mathematic form remains limited, which may also lead to probable inaccuracy to some extent. Finally, despite the lack of historical hydrologic data, a problem that is encountered by many studies all over the world, the likelihood of collecting as much catchment data as possible is still considered in constructing the regression model even though basic data collection is arduous and time-consuming.

## Conclusions

Flow velocity is a dynamic and essential parameter in GIUH-based models that are used to conduct runoff simulation in ungauged basins. The accuracy of the GIUH method largely relies on the calculation of the characteristic flow velocity estimation. However, the applicability of velocity calculation may be restricted due to non-availability of data, such as Manning’s roughness coefficient value and observed rainfall data, in traditional empirical approaches used in previous studies.

This study explored the numerical relationship between the characteristic flow velocity and the terrain and topographic parameters. This study also provided a new and simple perspective on GIUH generation without the need for observed hydrologic data. Moreover, this study aims

to improve the application of the GIUH method in runoff forecasting in ungauged watersheds or areas with limited data.

Equations that are based on the characteristic values of the geomorphologic and terrain structures are constructed by utilizing multivariate regression method, which can calculate the characteristic flow velocity of a basin. Thus, the GIUH can be generated. A total of 120 sub-basins in the Yangtze River Watershed are examined to ensure the availability and accuracy of the results.

A previous empirical method, which is also related to geomorphologic features, is adopted for comparison and analysis. The values of the evaluation criteria illustrate that the proposed regression model is suitable and applicable for practical flood simulation. Thus, the GIUH–*v*_*c*_ approach can be used for flood estimation with reasonable and good accuracy.

## Supplementary information

Basic geomorphologic characteristics of 120 watersheds
