# Supplementary material for: New method to calculate the dynamic factor–flow velocity in Geomorphologic instantaneous unit hydrograph
Source: Sci Rep. 2019 Oct 2;9:14201. doi: 10.1038/s41598-019-50723-x (PMC6775115; doi:10.1038/s41598-019-50723-x)
Supplement: Supplementary file 1 — Basic geomorphologic characteristics of 120 watersheds [file 41598_2019_50723_MOESM1_ESM.docx]

# Appendix I Basic geomorphologic characteristics of 120 watersheds

# Manuscript title: New method to calculate the dynamic factor–flow velocity in Geomorphologic instantaneous unit hydrograph

Author: Yingbing Chen^1^, Peng Shi^2*^, Xiaomin Ji^3^, Simin Qu^4^, Lanlan Zhao^5^, Fengcheng Dong^6^

| watershed | Basin area(km²) | Bifurcation ratio | Area ratio | Length Ratio | Length of Ω-order stream (m) | Length of longest stream (m) | Along-channel slope of Ω-order stream |
| --- | --- | --- | --- | --- | --- | --- | --- |
|  |  |  |  |  |  |  |  |
| **Calibration** | | | | | | | |
| Hetan | 434 | 4.41 | 4.56 | 2.15 | 20162 | 46977 | 0.00248 |
| Hongyanxi | 186 | 4.56 | 4.89 | 2.22 | 17733 | 31036 | 0.00237 |
| Jianshi | 159 | 4.82 | 4.92 | 2.05 | 18760 | 32835 | 0.00991 |
| Jinping | 493 | 4.26 | 4.35 | 2.08 | 19098 | 43157 | 0.00141 |
| Jiujietan | 106 | 4.98 | 5.57 | 2.51 | 18068 | 23567 | 0.0041 |
| Lianyuan | 158 | 4.32 | 4.4 | 2 | 10254 | 26184 | 0.00244 |
| Luolingqiao | 328 | 4.45 | 4.72 | 2.2 | 17008 | 35399 | 0.00076 |
| Maojiaqiao | 368 | 4.69 | 4.6 | 2.17 | 32435 | 53759 | 0.00182 |
| Putou | 188 | 5.04 | 4.85 | 2.09 | 23580 | 36062 | 0.00343 |
| Sankouzhen | 260 | 5.05 | 5.35 | 2.51 | 13860 | 40683 | 0.00159 |
| Shahebu | 462 | 4.76 | 4.9 | 2.31 | 16527 | 49432 | 0.00514 |
| Shenshan | 35.3 | 4.86 | 5.24 | 2.26 | 5726 | 11443 | 0.00227 |
| Shuangfengtan | 400 | 4.61 | 4.82 | 2.17 | 35680 | 44732 | 0.00645 |
| Tiantangyan | 348 | 5.11 | 5.21 | 2.26 | 39244 | 56297 | 0.01246 |
| Tieluping | 309 | 4.83 | 4.94 | 2.2 | 26844 | 53729 | 0.00652 |
| Tongcheng | 225 | 4.58 | 4.59 | 2.07 | 23775 | 47458 | 0.00139 |
| Wangcao | 401 | 4.8 | 4.82 | 2.1 | 13952 | 44682 | 0.00215 |
| Wuduhe | 205 | 4.76 | 4.53 | 1.95 | 21233 | 36950 | 0.01592 |
| Xiangshui | 485 | 4.4 | 4.52 | 2.17 | 12661 | 41164 | 0.00759 |
| Xiaohaizi | 59.7 | 4.71 | 5.1 | 2.43 | 4503 | 14418 | 0.01821 |
| Xinhua | 321 | 5.3 | 5.47 | 2.29 | 19089 | 37216 | 0.0231 |
| Xionglong | 104 | 5.1 | 5.28 | 2.52 | 12543 | 31288 | 0.00431 |
| Xiuwen | 201 | 4.73 | 4.85 | 2.35 | 8622 | 28948 | 0.00278 |
| Yinshan | 472 | 4.44 | 4.53 | 2.1 | 32912 | 59937 | 0.00188 |
| Yuantuo | 322 | 4.62 | 4.84 | 2.27 | 15179 | 43577 | 0.00072 |
| Zhonghe | 349 | 4.39 | 4.69 | 2.24 | 13516 | 46754 | 0.0037 |
| Ziyuan | 470 | 4.65 | 4.9 | 2.24 | 22513 | 46288 | 0.00222 |
| Baiyan | 634.5 | 4.45 | 4.52 | 2.12 | 26984 | 59183 | 0.00452 |
| Baiquan | 562.5 | 4.54 | 4.72 | 2.17 | 27187 | 51091 | 0.00349 |
| Caoba | 703.5 | 4.97 | 5.18 | 2.26 | 24286 | 70499 | 0.00786 |
| Dagutai | 733 | 4.81 | 5 | 2.45 | 35615 | 66350 | 0.00752 |
| Dapulu | 623 | 4.57 | 4.79 | 2.27 | 14611 | 61554 | 0.00103 |
| Dibao | 512 | 4.79 | 5.03 | 2.26 | 11432 | 43603 | 0.00566 |
| Dongkou | 895 | 4.83 | 5.02 | 2.36 | 25289 | 52146 | 0.00656 |
| Fengzhou | 689 | 4.94 | 4.93 | 2.27 | 43505 | 78345 | 0.00538 |
| Guanyang | 947 | 4.63 | 4.66 | 2.14 | 53518 | 79144 | 0.00198 |
| Hongchun | 884 | 4.95 | 4.89 | 2.18 | 41020 | 74430 | 0.00466 |
| Hufeng | 771 | 4.48 | 4.53 | 2.02 | 64984 | 90142 | 0.00069 |
| Huangmaocun | 736 | 4.44 | 4.73 | 2.33 | 49612 | 74747 | 0.00115 |
| Jishou | 775 | 4.47 | 4.64 | 2.23 | 34915 | 70154 | 0.00229 |
| Jindong | 798 | 4.83 | 4.98 | 2.37 | 33045 | 66282 | 0.00487 |
| Lichuan | 514 | 4.32 | 4.42 | 2 | 29152 | 56385 | 0.00082 |
| Linli | 742 | 4.73 | 4.8 | 2.26 | 33164 | 64012 | 0.00054 |
| Luodianhe | 738 | 4.85 | 5.1 | 2.3 | 21422 | 51540 | 0.01643 |
| Macheng | 886 | 4.86 | 5.07 | 2.41 | 34276 | 71510 | 0.00102 |
| Maliangping | 934 | 4.59 | 4.63 | 2.14 | 32300 | 63046 | 0.01576 |
| Mukong | 998 | 4.59 | 4.75 | 2.27 | 48437 | 77198 | 0.00188 |
| Pingshi | 751 | 5.03 | 5.31 | 2.47 | 24630 | 62940 | 0.00146 |
| Renli | 848 | 4.77 | 4.84 | 2.18 | 23255 | 66104 | 0.01853 |
| Shihaoqiao | 604 | 4.43 | 4.61 | 2.14 | 20207 | 48192 | 0.00114 |
| Shiqian | 739 | 4.75 | 4.99 | 2.25 | 12562 | 51726 | 0.00541 |
| Shuhe | 578 | 4.83 | 5.02 | 2.35 | 31474 | 55773 | 0.00702 |
| Wanyugou | 571 | 4.66 | 4.95 | 2.44 | 25504 | 55619 | 0.00514 |
| Bazhong | 2710.6 | 4.83 | 4.82 | 2.29 | 20400 | 131606 | 0.00123 |
| Baisha | 1589.2 | 4.45 | 4.51 | 2.25 | 54729 | 88288 | 0.00139 |
| Baitugang | 1051 | 4.83 | 4.94 | 2.29 | 69246 | 127897 | 0.00261 |
| Beitou | 1868.7 | 4.21 | 4.36 | 2.17 | 48054 | 92599 | 0.00062 |
| Chadianzi | 1699 | 4.82 | 5.03 | 2.46 | 13650 | 105927 | 0.00534 |
| Chaiping | 2363 | 4.68 | 4.83 | 2.27 | 80030 | 118419 | 0.00481 |
| Chishuihe | 3092 | 4.63 | 4.55 | 2.13 | 62652 | 136877 | 0.00632 |
| Dacaiyuan | 2202 | 4.54 | 4.67 | 2.27 | 84937 | 119406 | 0.00305 |
| Dafeng | 1129 | 4.53 | 4.6 | 2.16 | 40069 | 83026 | 0.00182 |
| Dahebian | 1274 | 4.52 | 4.69 | 2.18 | 47131 | 86623 | 0.00384 |
| Dazhuhe | 2648 | 4.5 | 4.55 | 2.1 | 58118 | 145079 | 0.00497 |
| Dongbeichong | 1674 | 4.58 | 4.84 | 2.33 | 29381 | 106112 | 0.00027 |
| Erlangba | 3183 | 4.58 | 4.73 | 2.14 | 53994 | 112751 | 0.00304 |
| Gaotan | 1075 | 4.53 | 4.54 | 2.21 | 36819 | 89394 | 0.00095 |
| Guanyin | 1894 | 4.54 | 4.65 | 2.25 | 56542 | 131631 | 0.00009 |
| Hongqi | 1321 | 4.68 | 4.57 | 2.03 | 56366 | 82554 | 0.0176 |
| Huangjinkou | 1260 | 4.69 | 4.8 | 2.15 | 57079 | 96943 | 0.00413 |
| Jiayi | 1562 | 4.52 | 4.67 | 2.29 | 33703 | 84339 | 0.0005 |
| Jiangbin | 2478 | 4.56 | 4.69 | 2.18 | 81105 | 132371 | 0.00197 |
| Laifeng | 1713 | 4.54 | 4.78 | 2.31 | 17921 | 107914 | 0.00241 |
| Lianghekou | 2815 | 4.74 | 4.73 | 2.2 | 69161 | 125804 | 0.00487 |
| Maoba | 1391 | 4.61 | 4.31 | 2.03 | 43601 | 110460 | 0.00227 |
| Neixiang | 1614 | 4.91 | 5.07 | 2.44 | 57186 | 119805 | 0.00171 |
| Ningnan | 3063 | 4.51 | 4.46 | 2.05 | 48216 | 145988 | 0.00738 |
| Qingfeng | 2050 | 4.59 | 4.78 | 2.36 | 32595 | 84262 | 0.0046 |
| Qionglai | 1424 | 4.68 | 4.98 | 2.43 | 16684 | 92979 | 0.00216 |
| Shazilin | 1236 | 4.63 | 4.83 | 2.27 | 51643 | 68818 | 0.00292 |
| Shanxiqiao | 1161 | 4.73 | 4.8 | 2.35 | 52631 | 85781 | 0.00549 |
| Shebu | 1406 | 4.61 | 4.91 | 2.32 | 49195 | 87801 | 0.00045 |
| Shibantang | 1621 | 4.56 | 4.83 | 2.32 | 33331 | 96158 | 0.00399 |
| Shimenkan | 1028 | 4.51 | 4.75 | 2.23 | 66778 | 96340 | 0.00097 |
| Sunshuiguan | 1584 | 4.85 | 4.8 | 2.11 | 56315 | 97909 | 0.00765 |
| **Validation** | | | | | | | |
| Anqiao | 250.5 | 4.82 | 5.04 | 2.21 | 26561 | 41814 | 0.003915 |
| Bainiqiao | 210.2 | 5.21 | 5.39 | 2.46 | 18357 | 41785 | 0.00196 |
| Cangping | 415.3 | 4.54 | 4.89 | 2.26 | 26151 | 56179 | 0.00348 |
| Binzhou | 361 | 4.46 | 4.5 | 2.05 | 19689 | 46664 | 0.00183 |
| Chuzhou | 287 | 4.73 | 4.68 | 2.06 | 34297 | 43668 | 0.01338 |
| Dawuchang | 148 | 4.05 | 4.44 | 2.11 | 3951 | 22109 | 0.0081 |
| Dongzhi | 466 | 4.54 | 4.91 | 2.36 | 17882 | 51289 | 0.0014 |
| Fengtun | 184 | 4.5 | 4.8 | 2.28 | 20608 | 37349 | 0.01266 |
| Fuquan | 63.7 | 4.7 | 5.04 | 2.27 | 8812 | 21833 | 0.01124 |
| Gushan | 321 | 4.51 | 4.63 | 2.16 | 14228 | 42585 | 0.00394 |
| Gulin | 374 | 4.32 | 4.54 | 2.01 | 24522 | 34666 | 0.02128 |
| Haofu | 432 | 4.35 | 4.68 | 2.07 | 14272 | 42876 | 0.00224 |
| Weifang | 973 | 4.51 | 4.69 | 2.17 | 39691 | 75246 | 0.00066 |
| Wugouxi | 857 | 4.67 | 4.74 | 2.25 | 15365 | 70919 | 0.00306 |
| Wuguan | 724 | 4.7 | 4.91 | 2.33 | 29900 | 75152 | 0.00823 |
| Xianrenzhang | 525 | 4.44 | 4.55 | 2.06 | 30683 | 60119 | 0.0016 |
| Xianhekou | 776 | 4.71 | 4.78 | 2.23 | 63907 | 87513 | 0.00466 |
| Xiushan | 652 | 4.67 | 4.96 | 2.31 | 40002 | 75974 | 0.00207 |
| Yanba | 592 | 5.2 | 4.78 | 2.22 | 26259 | 52999 | 0.00278 |
| Yanta | 804 | 4.58 | 4.83 | 2.29 | 30672 | 55564 | 0.00114 |
| Yangxinjiang | 561 | 4.59 | 4.89 | 2.3 | 30853 | 53440 | 0.00165 |
| Zhoujiahe | 541 | 4.53 | 4.51 | 2.09 | 23461 | 50810 | 0.00315 |
| Suoshi | 1648 | 4.48 | 4.59 | 2.03 | 45140 | 87631 | 0.01167 |
| Tianquan | 1719 | 4.5 | 4.51 | 2.09 | 38385 | 88349 | 0.0092 |
| WangCang | 1811 | 4.86 | 4.81 | 2.2 | 18888 | 132521 | 0.00344 |
| Wenquan | 1270 | 4.38 | 4.34 | 1.99 | 43188 | 85640 | 0.0144 |
| Wenxian | 7289 | 4.56 | 4.62 | 2.12 | 161444 | 234809 | 0.00951 |
| Wuxi | 2079 | 4.57 | 4.52 | 2.02 | 51271 | 110631 | 0.00616 |
| Wujiayuanzi | 1118 | 4.47 | 4.57 | 2.15 | 19894 | 66556 | 0.00281 |
| Xixiang | 1396 | 4.49 | 4.56 | 2.05 | 42209 | 94312 | 0.00211 |
| Xiaojia | 3159 | 4.57 | 4.53 | 2.29 | 112693 | 237851 | 0.00083 |
| Xingshan | 1929 | 4.61 | 4.84 | 2.24 | 12094 | 75521 | 0.00422 |
| Changtan | 1999 | 4.76 | 4.85 | 2.21 | 62141 | 132739 | 0.00605 |
| Zhuosang | 3057 | 4.4 | 4.5 | 2.13 | 107235 | 170158 | 0.00437 |
| Zongguantian | 1981 | 4.51 | 4.7 | 2.2 | 27663 | 88908 | 0.00777 |
